# The *Ginevra de’ Benci Effect*: Competence, Morality, and Attractiveness Inferred From Faces Predict Hiring Decisions for Women

**DOI:** 10.3389/fpsyg.2021.658424

**Published:** 2021-05-13

**Authors:** Michela Menegatti, Sara Pireddu, Elisabetta Crocetti, Silvia Moscatelli, Monica Rubini

**Affiliations:** Department of Psychology, Alma Mater Studiorum University of Bologna, Bologna, Italy

**Keywords:** sex discrimination, impression formation, facial traits, personnel selection, morality, competence, attractiveness

## Abstract

The present study examined the role of morality, competence, and attractiveness as perceived from faces in predicting hiring decisions for men and women. Results showed that for both female and male applicants, facial competence significantly predicted the hiring decision directly and indirectly, through the mediation of the overall impression. Decisions concerning female applicants were, however, significantly predicted by multiple dimensions—that is, facial morality, facial competence, and attractiveness—with the mediation of the overall impression. Facial competence was the only significant predictor of impression and, in turn, hiring decision about men. These findings resonate the motto *Virtutem forma decorat*, “Beauty adorns virtue,” painted by Leonardo da Vinci on the reverse side of the portrait of Ginevra de’ Benci, and suggest that women’s chances of getting a job are less than those of men whenever they do not show a moral *and* competent *and* attractive face.

## Introduction

**FIGURE 1:**
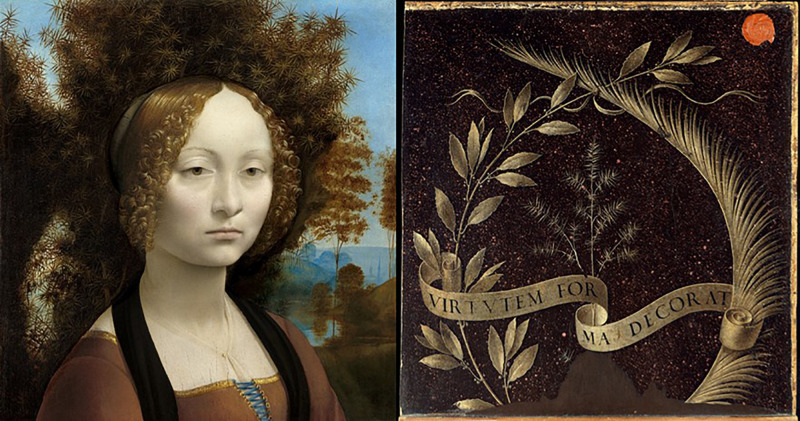
Leonardo da Vinci’s portrait of Ginevra de’ Benci and the reverse side of the painting.

Five centuries ago, Leonardo da Vinci painted the portrait of Ginevra de’ Benci, a Florentine noblewoman, probably on occasion of her wedding. Noteworthy is the reverse side of the portrait, which reveals Leonardo’s view of women: A ribbon bearing the motto *Virtutem forma decorat*, “Beauty adorns virtue,” binds a sprig of juniper (in Italian, *ginepro*), which evokes Ginevra’s name, whereas the encircling laurel and palm symbolize her intellectual and moral virtue (Figure 1). This representation suggests an inextricable relation between beauty, integrity, and intelligence in judging the worth of a woman. At the same time, it highlights the crucial importance of her facial appearance in making this judgment. Does Leonardo’s view of women apply even to the current time, when women look for a job (instead of a husband)? Do people consider these qualities all important when evaluating and making decisions about women’s career?

This research aims to address these questions by focusing on the effects of trait inferences from candidates’ faces in the context of personnel selection, whereby women still face unequal treatment as compared with men (European Commission, 2019; World Economic Forum, 2020). Accordingly, basing on recent research showing that female candidates are evaluated on more criteria than men (Prati et al., 2019; Moscatelli et al., 2020), we tested the contention that multiple dimensions—i.e., morality, competence, and attractiveness—would predict overall impressions and hiring decisions about female applicants. In contrast, facial competence might be the only important trait used to evaluate male applicants.

### Gender Inequality at Work and the “Perfection Bias”

Despite recent policies and efforts aimed at implementing non-discriminatory employment evaluations, many implicit ways through which job inequality in favor of men are still effective (e.g., Heilman and Eagly, 2008; Bruckmüller et al., 2013; Rubini and Menegatti, 2014; Ryan et al., 2016). Most of them are rooted in gender stereotypes (e.g., Heilman, 2012; Ellemers, 2018; Eagly et al., 2019; Hentschel et al., 2019), which depict men as higher in competence or agency, that is, more assertive, intelligent, and able; whereas women are seen as warmer or more communal, that is, more caring, helpful, and trustworthy (Wood and Eagly, 2002; Fiske et al., 2007, for the trait contents of agency and communion, see Abele et al., 2008). Interestingly, the negative correlation between the two dimensions implies that women, who are perceived as more communal, are also perceived as lacking competence and agency (Abele et al., 2008). This very likely depends on the autonomy connotation underlying the agency dimension in contrast to the dependence connotation of communality (Bakan, 1966). The direct consequence is a perceived lack of fit between the requirements of high-status job positions and the characteristics attributed to women. In turn, this perception leads to negative expectations about women’s performance in these jobs while relegating them to caring activities and domestic roles (Eagly et al., 1992; Heilman, 2012). Gender inequality in the workplace is further heightened by the fact that individuals with more agentic self-concepts are more successful in their occupational roles and careers, and career success in turn increases agentic traits (Abele, 2003) in a sort of virtuous circle that turns out to be vicious for women.

Another way through which gender stereotypes enhance gender-biased evaluations is related to the different standards used when making decisions about female and male candidates. Evaluators tend to set lower minimum standards of competence for women in the first phases of decision making, but at later stages, they ask women to provide even more evidence of competence to achieve the same outcome as their male counterparts (Biernat and Fuegen, 2001; Levin et al., 2005). Moreover, women who show competence and assertiveness are likely to be viewed as weak in social skills, and this, in turn, can negatively impact selection and career advancement (e.g., Rudman and Glick, 2001; Cuddy et al., 2004; Phelan et al., 2008).

Recent research (Prati et al., 2019; Moscatelli et al., 2020) took a somewhat different approach and examined gender inequality in personnel selection by considering the role of multiple dimensions at the basis of social judgment. These studies are grounded on a model (Leach et al., 2007) according to which the warmth dimension includes two distinct facets: morality, which refers to being benevolent to people in ways that facilitate correct relations with them (i.e., being honest, trustworthy, fair, and loyal), and sociability, which concerns individuals’ ability to establish and maintain good relationships with others (e.g., being nice and kind) (Leach et al., 2007; Abele et al., 2016; Crocetti et al., 2019; Brambilla et al., 2021). Building on this multidimensional model, Prati et al. (2019) examined spontaneous language use to refer to competence, morality, and sociability in descriptions made by professional committees evaluating female and male employees’ work performance. Results showed that professional selectors explained performance appraisals concerning male employees mainly on the ground of positive competence-related qualities, whereas morality and sociability were mentioned more often in reports of performance appraisals concerning female employees. Similar evidence was found by Moscatelli et al. (2020) by examining written reports of professional selectors, who relied on a wider range of qualities in their evaluations of female compared with male candidates. In a similar vein, when naïve evaluators were asked to rate the importance of a series of traits to hire a female or a male candidate, competence, morality, and sociability were all considered important to evaluate female candidates, whereas competence was considered the most important quality that men should have. The results of these studies converged in showing a “perfection bias” against women, according to which female candidates are evaluated on more qualities than male candidates. In further studies (Moscatelli et al., 2020), participants were asked to evaluate fictitious candidates who were presented as high vs. low in morality and competence. Sociability was not considered because it emerged as the least important criterion of judgment in the previous studies. Findings demonstrated that hiring and retention decisions concerning male candidates were predicted primarily by candidates’ (high or low) level of competence. Decisions on female candidates were instead predicted by the dimension along which they appeared to be weaker, being that competence or morality. This effect has been explained in terms of the aforementioned perfection bias: if greater importance is attributed to multiple criteria in evaluation of women, then a weakness on a single dimension is more likely to influence the final decision.

Overall, this line of research provides new insights into the processes underlying biased gender evaluation in personnel selection by demonstrating a systematic tendency to evaluate women on more traits than men. However, the role of facial appearance in selection processes remained unexplored. Conversely, it is well established that appearance does matter (Zebrowitz and Montepare, 2008). Evaluators acquire information on candidates’ traits and characteristics not only from relatively objective information, such as their previous qualifications, work experience, or results on psychological tests but also from their looks (e.g., Shannon and Stark, 2003). Even professional recruiters assert of being able to draw conclusions on personality dimensions and behavior from applicants’ profile pictures (Caers and Castelyns, 2011). These inferred traits, in turn, affect their willingness to invite candidates for job interviews (Baert, 2018). Thus, it is crucial to examine whether women are target of a perfection bias—namely, are evaluated on the ground of multiple dimensions—also when their personality traits are inferred from facial appearance.

### Trait Inferences From Faces and Gender Differences

People spontaneously and rapidly infer personal traits from faces. Less than 100 ms of exposure is enough to form impressions on others’ character (for a review, see Todorov et al., 2015). These inferences are pervasive, consensual, and linked to significant outcomes, such as voting preferences (e.g., Little et al., 2007; Olivola and Todorov, 2010), leadership selection and compensation (e.g., Rule and Ambady, 2008; Fruhen et al., 2015), and judicial decisions (e.g., Zebrowitz and McDonald, 1991). For instance, facial trustworthiness predicted the willingness to invest more money in others in strategic economic games (Rezlescu et al., 2012; Tingley, 2014), and defendants who have untrustworthy-looking faces were more likely to receive guilty verdicts (e.g., Dumas and Testé, 2006). Political candidates were more likely to win elections the more competent-looking their faces (e.g., Todorov et al., 2005; Castelli et al., 2009), and competent-looking CEOs were hired by more successful companies and received larger salaries (Rule and Ambady, 2009; Graham et al., 2016).

Overall, these findings showed that the influence of each facial trait on impression formation and decision making depends critically on its relevance to the domain in question. However, looking deeply into the existing literature, one might notice substantial differences in the relative importance of these judgments for women and men. For instance, trustworthy-looking faces were perceived as more feminine (Oosterhof and Todorov, 2008), whereas competent-looking faces were perceived as more masculine (Oh et al., 2019). Moreover, dominant female faces were judged more negatively than non-dominant female faces and dominant and non-dominant male faces (Sutherland et al., 2015). Interestingly, higher degrees of competence were attributed to persons with typically masculine faces than to persons with typically feminine faces, at least when decisions were made under controlled information processing (Sczesny and Kühnen, 2004).

Finally, when studying the effects of facial appearance on impression and hiring decisions about women and men, it is fundamental to consider the role played by applicants’ perceived attractiveness. According to the well-known “what is beautiful is good” effect, attractive individuals were believed to possess more socially desirable qualities (Eagly et al., 1991; Zebrowitz et al., 2002), which, in turn, elicited a preferential treatment of attractive people in a variety of domains, including the work context (Langlois et al., 2000; Zebrowitz, 2017). Attractiveness increased one’s chance of getting a job (Jawahar and Mattsson, 2005), the assessment of employee’s potential (Heilman and Stopeck, 1985; Marlowe et al., 1996), and the likelihood to obtain recommendations for salary raises and promotions (Heilman and Stopeck, 1985). However, beauty can be “beastly” for female candidates. Attractive women were considered as less qualified, were less likely to be recommended, and were perceived as deserving lower salaries when being evaluated for male-typed jobs (e.g., Cash et al., 1977; Heilman and Saruwatari, 1979). Recently, similar evidence has been found for feminine roles and work contexts, whereby attractiveness predicted less perceived truthfulness and more perceived deservingness of termination of female, but not male employees delivering negative organizational news (Sheppard and Johnson, 2019).

Notably, the research reported above mainly concerned the consequences of being—or not being—attractive for women and men. As far as we know, few studies have examined the relative weights of attractiveness and other facial traits in predicting gender-biased evaluations and decisions. For instance, in the context of voting preferences, facial competence was the strongest winning predictor for male politicians, whereas for women, the main predictor was attractiveness (Chiao et al., 2008; Poutvaara et al., 2009). Moreover, by analyzing pay decisions, Fruhen et al. (2015) found that attractiveness mainly predicted the pay awarded to lower-level managers, whereas facial trustworthiness and dominance were the main predictors of the reward attributed to senior managers. Interestingly, all these features were more predictive of female than male managers’ pay, suggesting that facial appearance had a stronger impact on personnel decisions concerning women than men.

Thus, people form consensual impressions from others’ faces on several specific dimensions, especially trustworthiness and competence (Todorov et al., 2015). Moreover, they use attractiveness as an important cue to decide on a persons’ value (Zebrowitz and Montepare, 2008). At the same time, however, how these facial traits are used to make decisions about others dramatically varies from women to men in diverse domains (e.g., Eagly et al., 1991; Fruhen et al., 2015; Oh et al., 2019). Given that professional recruiters increasingly use social media sites to look for information and form impressions about job applicants, the gender-related effects of facial first impression reviewed above could play an even more significant role in the hiring process. To address this issue, the present study examined whether facial traits inferred from faces have different weights in predicting personnel evaluations about female and male job candidates. In doing so, we tested the idea that female applicants would be the target of a perfection bias at the level of facial appearance and, therefore, would be evaluated on more facial traits than men would.

## Overview

The present study aims to examine whether the tendency to evaluate women along more dimensions than men (Prati et al., 2019; Moscatelli et al., 2020) could be detected when selection judgments are made on the ground of candidates’ facial appearance. To this aim, we analyzed the combined effects of morality, competence, and attractiveness inferred from candidates’ faces on impressions and hiring decisions concerning female and male candidates for a job position.

Research on facial first impression has been mainly focused on trustworthiness judgments from faces, without considering that trustworthiness is a crucial component of the broader morality dimension (e.g., Leach et al., 2007; Abele et al., 2016, 2008). To fill this gap and basing on evidence showing that morality is the primary dimension in impression formation (Wojciszke and Abele, 2008; Goodwin et al., 2014; Menegatti et al., 2020), in the present study, we chose to test the effects of *morality* inferred from candidate faces on impression formation and decision making. On its part, *competence* is the most important trait when judging whether a person is suitable for a job (Brambilla et al., 2011; Pagliaro et al., 2013; Prati et al., 2019; Moscatelli et al., 2020), and facial competence is the main predictor of important outcomes in the work context (e.g., Todorov et al., 2005; Rule and Ambady, 2008). Finally, we considered the effects of *attractiveness*, which, as mentioned, is a decisive predictor of positive evaluations in personnel selection (Zebrowitz and Montepare, 2008) and has quite different consequences for women and men (e.g., Heilman and Saruwatari, 1979; Sheppard and Johnson, 2019).

In the present study, participants were presented with CVs and photos of eligible candidates for a position that was perceived as equally suitable for women and men. This choice was made to test our predictions beyond the possible effects of the lack of fit (Heilman, 2012) between candidates’ perceived traits and those stereotypically expected to fill the job position. Participants were then asked to rate each candidate’s morality, competence, and attractiveness, and to evaluate their likelihood of being selected for the job.

Based on previous evidence showing a perfection bias against women (Prati et al., 2019; Moscatelli et al., 2020), we expected that all the traits considered—i.e., morality, competence, and attractiveness—perceived from candidates’ faces would significantly predict the overall impression of female candidates, which, in turn, should predict the final hiring decision. Conversely, given the prominent role of competence in evaluations of men (Fiske et al., 2007; Ellemers, 2018; Hentschel et al., 2019), competence inferred from faces should be the only significant predictor of impressions, and subsequent hiring decisions, concerning male applicants.

## Materials and Methods

### Participants

Participants were 221 students (106 females; seven participants did not report their gender; *M*_*age*_ = 21.44, *SD*_*age*_ = 3.14) of the University of Bologna, who completed the questionnaire at the end of classes. Half of them evaluated female applicants (*n* = 110) and the other half male applicants (*n* = 111). It is commonly recommended that structural equation models (SEMs) incorporating latent variables require a sample size of at least 200 participants to be accurate or that the ratio for sample size to estimated parameters should be 5:1 (Kelloway, 2015). Given that our model has 36 parameters (64 for multigroup analyses), we collected a sample size larger than 200 and in between the two ratios.

### Procedure and Material

The study has been approved by the Bioethics Committee of the University of Bologna. Before filling in the questionnaire, participants signed a consent form in line with the ethical norms of the University. Participants were presented with a paper and pencil questionnaire and asked to “imagine being members of the Teaching Board of their Department, which, by statute, is composed of equal numbers of professors and students. The Board had to select a student (“the applicant”) for a temporary, part-time position in the administrative office. The tasks of the job were data entry, making copies of teaching material, managing reservation of teaching and meeting rooms, administering the website.” This position was chosen because student participants were likely to know it better than most non-academic jobs, and it is considered equally fitting for men and women. A pretest with 34 university students (20 females; *M*_*age*_ = 22.68, *SD*_*age*_ = 2.57) supported that this position was perceived as similarly suitable (1 = *not at all*; 7 = *very much*) for women (*M* = 4.98, *SD* = 1.06) and men (*M* = 4.97, *SD* = 1.06), *t*(33) = 0.10, *p* = 0.919, 95% CI = [-0.13, 0.14]. In the subsequent pages of the questionnaire, participants were asked to evaluate an applicant basing on the brief CV that s/he sent. The CV was the same for all applicants and reported basic information in Europass format: name, age 21, Italian nationality, undergraduate student of the Department of Psychology, marks average 27/30, English level B1, and good digital competence with Office package. We did not provide information about applicant’s sexual orientation. A photo of the applicant was attached to the top-left corner of the CV. The photos depicted faces with neutral expressions that were retrieved from the Karolinska Directed Emotional Faces (Lundqvist et al., 1998). We selected 16 photos (eight females and eight males) distributed along different levels of trustworthiness (which fall into the morality and competence dimensions; Leach et al., 2007), intelligence (a component of the competence dimension), and attractiveness, basing on the scores attributed to the faces in Oosterhof and Todorov’s (2008) study. See the Supplementary Material for the selected photos. All the persons depicted in the photos wore a gray t-shirt with no jewelry, piercings, or other marks that could affect evaluation judgments.

After reading the CV, participants were asked to rate (1 = *not at all*; 7 = *very much*) to what extent the person portrayed in the photo looked “honest” and “moral” (facial morality), “intelligent” and “competent” (facial competence), and “good-looking” and “attractive” (facial attractiveness). To assess perceived morality, we did not use the term “trustworthy” because its meaning, in the Italian translation (*affidabile*), is closer to that of “reliable” and can be intended as more related to competence than morality, especially in the job context. In contrast, “honest” is undoubtedly a trait of the moral domain (e.g., Brambilla et al., 2011). Then, we measured participants’ global impression on each applicant asking, “Which is your impression about the candidate?” with 1 indicating a negative impression and 7 a positive impression (Wojciszke et al., 1998). Participants’ hiring decision was measured by means of two items adapted from Rudman and Glick (1999): “In your opinion, how likely is it that the applicant would be selected for the job?” and “Would you select the applicant?” (1 = *very unlikely*; 7 = *very likely*). Finally, participants filled in their demographic information.

## Results

The dataset is publicly available at https://osf.io/6y4zk/

### Preliminary Analyses

We first average inferences made on “honest” and “moral” items to obtain a facial morality score, *r*(219) = 0.65, *p* < 0.001, “competent” and “intelligent” items to obtain a facial competence score, *r*(220) = 0.58, *p* < 0.001, and “good-looking” and “attractive” items to obtain a facial attractiveness score, *r*(219) = 0.80, *p* < 0.001. We also averaged answers to the two items measuring participants’ hiring decision, *r*(219) = 0.79, *p* < 0.001. Then, as a preliminary step, we tested whether morality, competence, and attractiveness inferred from faces can be considered, in line with the model of Leach et al. (2007; see also Brambilla et al., 2021) as distinct dimensions of impression formation. To this end, we conducted a confirmatory factor analysis in M*plus* 8.1 (Muthén and Muthén, (1998-2017)), using the maximum likelihood robust (MLR) estimator (Satorra and Bentler, 2001). We tested a model whereby the dimensions (morality, competence, and attractiveness) underlying trait inferences from faces were represented by three latent variables (with two observed indicators each). We evaluated the model fit by means of multiple indices (Byrne, 2012): the comparative fit index (CFI) and the Tucker–Lewis index (TLI), with values higher than 0.90 indicative of an acceptable fit and values higher than 0.95 suggesting an excellent fit; and the root mean square error of approximation (RMSEA), with values below 0.08 indicative of an acceptable fit and values less than 0.05 representing a very good fit. In addition, we inspected the 90% confidence interval of the RMSEA (when the upper bound of this confidence interval is ≤ 0.10, the model fit can be considered acceptable; Chen et al., 2008) and the Akaike information criterion (AIC), according to which the model with the smallest AIC is the best-fitting one. Results are reported in Table 1 and showed that the model with three latent factors has unequivocally better fit indices than the models with one or two latent factors. Descriptive statistics and correlations among variables are reported in Table 2.

**TABLE 1:** Confirmatory factor analyses comparing models with three, two, and one latent factors.

	**CFI**	**TLI**	**RMSEA [90% CI]**	**AIC**
**Three-factor model**	1.000	1.002	0.000 [0.000, 0.085]	3,746.420
Morality				
Competence				
Attractiveness				
**Two-factor model**	0.816	0.654	0.205 [0.166, 0.246]	3,824.253
Morality				
Competence and attractiveness				
combined				
**Two-factor model**	0.769	0.567	0.229 [0.190, 0.270]	3,847.742
Competence				
Morality and attractiveness				
combined				
**Two-factor model**	0.771	0.570	0.228 [0.190, 0.269]	3,819.894
Attractiveness				
Morality and competence				
combined				
**One-factor model**	0.610	0.350	0.281 [0.244, 0.319]	3,916.232

**CFI, comparative fit index; TLI, Tucker–Lewis index; RMSEA, root mean square error of approximation; AIC, Akaike information criterion.**

**TABLE 2:** Means, standard deviations, and correlations among all study variables.

	***M***	***SD***	**2**	**3**	**4**	**5**
1. Morality	4.14	1.03	0.30***	0.31***	0.40***	0.26***
2. Competence	4.55	0.98	–	0.32***	0.60***	0.57***
3. Attractiveness	2.66	1.27		–	0.48***	0.36***
4. Impression	4.16	1.10			–	0.71***
5. Hiring decision	4.07	1.51				–

***p < 0.001.

### Trait Inferences, Impression, and Hiring Decision

In order to test whether traits inferences from faces, impression, and hiring decisions were comparable for female and male applicants and participants, we conducted a series of 2 (participant gender) × 2 (applicant gender) univariate ANOVAs. The analysis on perceived morality showed no significant effects, all *F*s < 1.70, *p*s > 0.193. With respect to perceived competence, there was the main effect of participant gender, with female participants inferring less competence from candidates’ faces (*M* = 4.34, *SD* = 0.97) than male participants (*M* = 4.74, *SD* = 0.95), *F*(1, 209) = 9.06, *p* = 0.003, η_*part*_^2^ = 0.042. The other effects were not significant, *F*s < 0.857, *p*s > 0.356. Attractiveness was perceived as higher for female (*M* = 2.94, *SD* = 1.30) than for male candidates (*M* = 2.34, *SD* = 1.15), *F*(1, 209) = 12.55, *p* < 0.001, η_*part*_^2^ = 0.057. There were no other significant effects, *F*s < 1.19, *p*s > 0.277.

The ANOVA on the global impression about the candidate, all *F*s < 3.13, *p*s > 0.078, and that on hiring decision, all *F*s < 3.04, *p*s > 0.083, showed no significant effects.

### Structural Equation Modeling Analyses

To address the main aim of the Study, we conducted SEM analyses whereby the dimensions underlying trait inferences from faces, namely, morality, competence, and attractiveness, were represented by three latent variables (with two observed indicators each) and predicted the hiring decision, represented by one latent variable (with two observed indicators), both directly and indirectly, through the mediation of overall impression (represented by one observed variable). We also included in the model correlations between the three dimensions inferred from faces. We tested the model in two independent groups, defined on the basis of applicant gender, after having confirmed measurement invariance (see Supplementary Material for analyses and results). As discussed above, we evaluated the model fit by means of multiple indices (CFI, TLI, and RMSEA).

**FIGURE 2:**
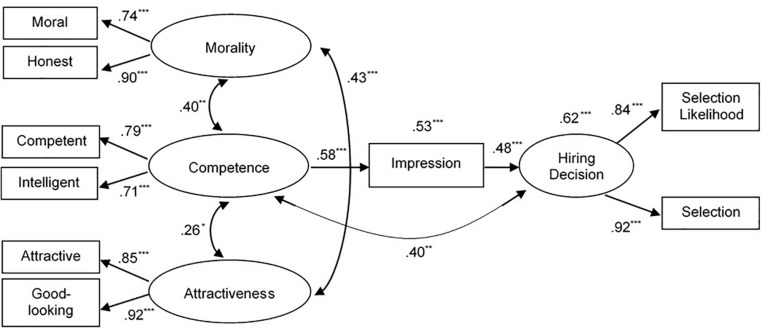
Structural equation model for male applicants. Values reported above impression and hiring decision indicate portions of explained variance. *p < 0.05; **p < 0.01; ***p < 0.001.

**FIGURE 3:**
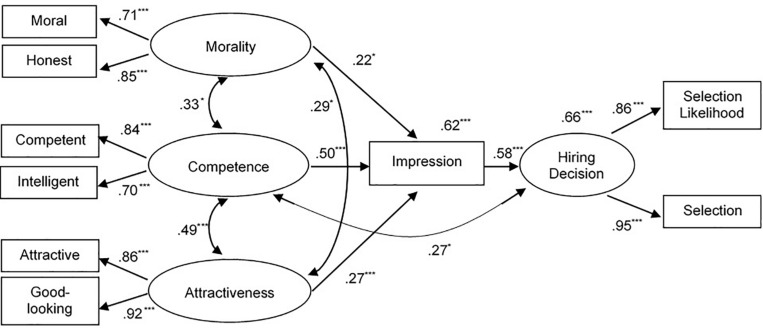
Structural equation model for female applicants. Values reported above impression and hiring decision indicate portions of explained variance. *p < 0.05; **p < 0.01; ***p < 0.001.

The results of the multigroup analyses indicated that the model tested in the two separate groups fitted the data very well, χ_*SB*_^2^ = 50.417, df = 44, *p* = 0.235, CFI = 0.993, TLI = 0.988, RMSEA = 0.036 [0.000, 0.076]. Standardized parameter estimates are reported in Figure 2 for male applicants and Figure 3 for female applicants. In line with the hypotheses, we found meaningful differences based on applicants’ gender. Specifically, for male applicants, only facial competence was significantly related to hiring decision, both directly and indirectly, by positively affecting overall impression, which in turn was strongly and positively related to hiring decisions, β = 0.28, *p* < 0.001, 95% CI = [0.15, 0.42]. There were no direct nor indirect effects of facial morality, β = 0.06, *p* = 0.246, 95% CI = [-0.04, 0.15], and attractiveness, β = 0.10, *p* < 0.107, 95% CI = [-0.02, 0.21] on hiring decision. In contrast, for female applicants (Figure 2), all indirect effects were statistically significant: facial morality, β = 0.13, *p* = 0.039, 95% CI = [0.01, 0.25], facial competence, β = 0.29, *p* < 0.001, 95% CI = [0.15, 0.43], and facial attractiveness β = 0.16, *p* = 0.007, 95% CI = [0.04, 0.27]. In other words, all traits inferred from female applicants’ faces were significantly related to the overall impression, which in turn significantly mediated the effects of trait inferences on hiring decisions. In addition to these indirect effects, a direct effect of competence on hiring decisions was detected. Notably, the percentages of explained variance were high for both overall impression (62 and 53% for female and male applicants, respectively) and hiring decisions (66 and 62% for female and male applicants, respectively). As ancillary analyses, we used Wald test to examine whether the strengths of the paths from morality, competence, and attractiveness to impression were significantly different for male and female applicants. Results indicated that the paths for male and female applicants did not differ for morality (Wald test = 0.716, df = 1, *p* = 0.397), competence (Wald test = 0.007, df = 1, *p* = 0.934), and attractiveness (Wald test = 0.286, df = 1, *p* = 0.593). Nonetheless, for male applicants, only competence significantly predicted the overall impression, whereas for female applicants, all the dimensions considered—morality, competence, and attractiveness—significantly predicted impression formation.

Overall, these findings showed that for both women and men, facial competence directly predicted the hiring decision. Moreover, competence inferred from faces was the only predictor of impression and, in turn, hiring decision on male applicants, whereas all the dimensions considered predicted the hiring decision on female applicants, with the mediation of the overall impression.

## General Discussion

We opened this paper by wondering whether Leonardo’s idea of women could be applied even in contemporary societies. Our findings could suggest an affirmative answer. By examining the relative importance of morality, competence, and attractiveness perceived from faces in predicting job evaluations, we found that multiple traits inferred from applicants’ faces—i.e., morality, competence, and attractiveness—significantly affected impressions and hiring decisions about female applicants. Conversely, a single dimension, facial competence, significantly predicted impressions and decisions about male applicants. This means that, in the work context, facial competence remains a key dimension to evaluate both women and men (see Brambilla et al., 2011; Moscatelli et al., 2020), but every single dimension made an independent and significant contribution to form impressions and making decisions about female candidates. These findings are consistent with recent research on the “perfection bias” (Prati et al., 2019; Moscatelli et al., 2020) and suggested that impression formation and decision-making processes about women involve a configuration of multiple traits attributed to the person based on her facial appearance, whereas less information is used in the case of male candidates. The direct consequence is that looking competent could be enough for men to be hired, whereas women should look as moral *and* competent *and* attractive, or, to put it differently, as “apparently perfect.” The present study further showed that these evaluations based on multiple criteria for women are made in a spontaneous and subtle way, just looking at the candidate’s face. Overall, this line of research converged to unveil one of the possible subtle mechanisms that might work at different levels and through different routes of the evaluation process to produce, maintain, and justify gender inequality in the workplace by making the hiring process more demanding for women.

### Implications for Theory and Research

The present study goes beyond previous research on gender discrimination in the workplace in several important ways. First, we considered a different set of judgment dimensions than those traditionally examined by research on gender stereotypes, which has mainly contrasted warmth and competence stereotypic expectations to explain biased job evaluations (for reviews, see Heilman, 2012; Ellemers, 2018). This choice allowed us to uncover a further implicit process through which women’s access to employment and career might be hindered. Precisely, we highlighted that women are evaluated on multiple—and not only different—standards than men and that evaluations can be strongly affected by traits inferred merely by their facial characteristics as they contribute to the overall process of impression formation. A possible implication of this bias is that appearing little moral or little attractive should be quite irrelevant for male candidates. On the contrary, if women fail to show either a moral or a competent or an attractive face, they could be judged less favorably, and their chances to be hired are likely to decrease dramatically. Future research could test this possibility by using morphing methods (see Sutherland et al., 2016), which allow to experimentally vary facial cues and obtain faces with different and controlled levels of morality, competence, and so on. This should allow to examine, for example, the specific effect of high vs. low attractiveness on impressions and decisions by keeping constant the other dimensions.

Moreover, we uncovered for the first time the specific role of morality inferred from facial cues in judgments about women. Previous research has demonstrated that this dimension has a primary role in forming impressions about others (e.g., Brambilla et al., 2021) and deciding on how to behave toward them (e.g., Brambilla et al., 2016; Prati et al., 2018; Menegatti et al., 2020) and that trustworthiness is the key personal trait inferred from others’ faces (e.g., Todorov et al., 2015). Our study further highlighted that facial morality did contribute to form impressions of and make judgments about women but not men. Similar considerations could be made for attractiveness, which significantly predicted evaluations of female but not male applicants. This suggested that the “what is beautiful is good” effect (Eagly et al., 1991) might mainly concern women and that the less attractive women are, the less their likelihood of being selected at least for a not gender-typed position as the one considered here. The story is quite different for men, as their level of attractiveness does not seem to affect their chances of being hired. In this respect, it should be stressed that considering a not gender-typed job—as we did in this study—allowed to conclude that the differences in evaluation of women and men that we found did not depend on a perceived lack of fit between gender stereotypic traits and the characteristics thought to be necessary for masculine (e.g., leadership) positions (e.g., Davison and Burke, 2000; Heilman, 2012).

Our findings suggest the presence of a more general bias that could hinder women’s professional achievements independently from the type of job (for similar reasoning, see also Sheppard and Johnson, 2019). Namely, women might be the target of a perfection bias at the level of facial appearance: they are evaluated not only on their facial competence but also on additional facial cues. This might represent a powerful yet implicit mechanism that contributes to bias employment decisions.

### Practical Implications

The present study highlighted a novel way through which facial appearance can implicitly contribute to gender inequality in evaluations and decisions about job candidates. We are aware that the personnel selection process is not exclusively based on facial appearance and that gender inequality is a result of more complex processes. However, it is now demonstrated that evaluators, especially under time pressure and cognitive load, rely on judgmental shortcuts, easily available cues, and lay theories that are misperceived to be predictive of job performance (e.g., Florea et al., 2019). For this reason, early impressions from facial appearance would very likely affect the final hiring decision. As mentioned, human resources professionals increasingly look for information about job candidates on social media sites, like Facebook and LinkedIn (Brown and Vaughn, 2011), and draw conclusions on personality dimensions and behavior from applicants’ profile pictures (Caers and Castelyns, 2011). This first impression, in turn, might influence recruiters’ behavior and information processing during the remaining phases of the assessment, so that the final hiring decision might coincide with the very first evaluation. Keeping in mind these considerations, our results might further suggest that how women’s face is perceived could make their career at least three times as difficult as the career of their male counterpart.

In light of these considerations, selectors should be trained to refrain from making personality trait inferences from candidates’ faces and avoid using different routes of evaluations for female and male candidates. Importantly, the practices of looking for candidates’ information in social media websites should be discouraged, and organizations should provide time, resource, information, criteria, and standards in order to avoid ambiguity (e.g., Heilman and Haynes, 2008) and thus decrease the possibility that selectors recur to evaluation shortcomings. Employers and recruiters should also be asked to document all information gathered and used in the evaluation process and to explicitly justify reasons for their decisions (Brown and Vaughn, 2011). These conditions are essential not only to contrast gender inequality in the workplace but also to relieve women from a further burden that they could feel when applying for a job. Indeed, female candidates could be aware that their facial appearance might affect the evaluation process without knowing how to contrast the possible negative effects of this bias. As a consequence, they could feel uneasy about showing their photo in the CV or being interviewed, and therefore, they could decide not to apply for certain jobs, especially those for which physical appearance is perceived to be more relevant. For all these reasons, and considering that the biasing impact of facial appearance is often mitigated (e.g., Lenz and Lawson, 2011; Rezlescu et al., 2012) but not eliminated by access to more relevant information (e.g., Olivola and Todorov, 2010), the effect we unfolded in the present study could represent a subtle and—precisely because of its implicit nature—powerful means through which women might be discriminated against.

### Limits and Future Directions

One of the major limits of the present study is that we used photos depicting faces with neutral expressions, whereas real-life candidates might, of course, display a variety of facial expressions in different phases of the selection process. We made this choice because it is well documented that emotion expressions strongly affect the perception of personality traits from faces (e.g., Todorov et al., 2015). This is especially true for trustworthiness since smiling faces are perceived as more trustworthy and frowning faces as less trustworthy and more dominant (Oosterhof and Todorov, 2008). Using neutral faces allowed to control for the influences of facial expressions on trait inferences, but future research might certainly examine whether our pattern of results can also be found when candidates are evaluated with more realistic photos.

It would also be crucial to test whether women and men candidates are evaluated on different criteria by adopting a different approach. Albeit we found that all the criteria were statistically significant for female candidates, whereas only competence was a significant predictor of impression for male candidates, ancillary analyses indicated that the strength of the paths obtained for female and male candidates did not differ significantly. This is possibly due to the specific design and the use of faces as stimuli. Indeed, the evaluative dimensions of morality, competence, and attractiveness refer to facial traits that are difficult to “isolate”: when we see a face, we build a general impression based on a configuration of cues rather than on a single facial characteristic. For this reason, future research should examine the relative weight of different facial traits for the evaluation of women and men by using a design with applicant gender as within-subjects factor. In particular, studies explicitly designed to force participants to compare the perception of female and male candidates on several dimensions—a procedure closer to real-life situations—might allow to definitively clarify whether, as we suggest basing on the present findings, hiring decisions concerning women are made on more criteria than men’s and also whether the relative weight of these dimensions differs as a function of candidate gender.

A further issue to be investigated is whether evaluations based on multiple facial traits resulted in a real disadvantage for women. Indeed, we did not find significant differences in hiring decisions between female and male candidates. We believe that this is mostly due to the choice of using a position for which male and female candidates were considered equally suitable. Thus, it would be important to verify whether the facial traits of female and male candidates differently affect impressions and selection decisions for stereotypically feminine and masculine jobs.

A final limitation of the present research concerns the specific cultural context in which data have been collected. The gender gap is particularly pronounced in Italy, which has been warned by the European Committee of Social Rights (ECSR) about non-compliance with the right to equal pay and equal working opportunities for women and men. Thus, it would be worthy for future research to test whether our results could be generalized to cultures where gender discrimination in the workplace is lower than that of Italy. Related to this, it would also be possible that, in countries where women are more represented in certain jobs (specifically high-status professions), traits inferences from faces would have a different effect on impressions and hiring decisions than those we found here. Indeed, recent studies (e.g., Goodale et al., 2018; Lick and Johnson, 2014) have shown that judgments about individuals’ faces can be influenced by contextual information, specifically the base rate information of a group affects the categorization of members of that group. Based on this evidence, future research might examine whether manipulating the presence of women (high vs. low) in a particular job could change participants’ impressions based on the faces of women (but not men) who applied for that job.

## Conclusion

Despite decades of progress, factual gender equality in the workplace remains challenging to achieve. Explicit discriminations and injustices like the glass ceiling (Ryan et al., 2016) or the wage gap (European Commission, 2019; World Economic Forum, 2020) could represent just the tip of an iceberg with implicit biases in evaluations lurking below the surface. In our view, the present study contributes to identify one of the possible subtle processes at the source of these biases and thus be useful to eradicate them. All in all, our findings suggest that, as long as personnel selectors can rely on applicants’ pictures and face-to-face interviews, women’s employment opportunities might remain fewer than those of men, unless they appear as a contemporary Ginevra de’ Benci. Indeed, the motto *Virtutem forma decorat* painted in the reverse side of the portrait might, at a first sight, be considered as a favorable view of women. However, as models of benevolent sexism (Glick and Fiske, 2001) imply, portraying women in multiple positive terms can hinder the intentions of achieving factual and real gender equality in the workplace. While for Ginevra beauty, virtue, and intellect were the *sine qua non-conditions* for a high-society marriage, in the current time, evaluating women on multiple criteria might contribute to hinder the achievement of the same goals that are granted to men on the basis of the exclusive evaluation of their competence.

## Data Availability Statement

The datasets presented in this study can be found in online repositories. The names of the repository/repositories and accession number(s) can be found below: https://osf.io/6y4zk/.

## Ethics Statement

The studies involving human participants were reviewed and approved by Bioethics Committee of the University of Bologna. The patients/participants provided their written informed consent to participate in this study.

## Author Contributions

MM, SP, and SM conceived and designed the experiment. SP collected the data. EC conducted the statistical analyses. MM wrote the manuscript. MR supervised and funded the research. All authors contributed to the final version of the manuscript.

## Conflict of Interest

The authors declare that the research was conducted in the absence of any commercial or financial relationships that could be construed as a potential conflict of interest.
