# Supplementary material for: The Ginevra de’ Benci Effect: Competence, Morality, and Attractiveness Inferred From Faces Predict Hiring Decisions for Women
Source: Front Psychol. 2021 May 13;12:658424. doi: 10.3389/fpsyg.2021.658424 (PMC8155713; doi:10.3389/fpsyg.2021.658424)
Supplement: Supplementary file 1 [file Data_Sheet_1.docx]

**The Ginevra de’ Benci Effect: Competence, Morality, and Attractiveness Inferred from Faces Predict Hiring Decisions for Women (not for Men)**

**Supplementary Material**

**Stimulus Material: Faces**

Labels and scores attributed by Oosterhof and Todorov (2008) to trustworthiness, intelligence, and attractiveness traits of the 16 photos used for the present Study. The scores are standardized means. “AF” labels refer to female photos and “AM” to male photos (see the original study for further information).

|  | trustworthy | intelligent | attractive |
| --- | --- | --- | --- |
| **AM03NES** | -1,56 | -0,87 | -1,07 |
| **AM07NES** | -1,51 | -0,43 | -0,84 |
| **AM08NES** | 0,99 | 0,75 | 0,75 |
| **AM09NES** | -0,02 | 0,19 | -0,6 |
| **AM14NES** | 0,65 | 0,69 | 1,47 |
| **AM17NES** | -0,28 | 0,42 | -0,22 |
| **AM25NES** | -0,25 | 0,55 | -0,17 |
| **AM31NES** | 1,04 | 0 | 0,42 |

|  | trustworthy | intelligent | attractive |
| --- | --- | --- | --- |
| **AF03NES** | 0,22 | -0,61 | -0,19 |
| **AF11NES** | 0,62 | 0,75 | 1,43 |
| **AF13NES** | 0,77 | 0,63 | 1,18 |
| **AF15NES** | 0,18 | 0,31 | 0,41 |
| **AF18NES** | 0,11 | 0,68 | 0,3 |
| **AF24NES** | -0,14 | -0,41 | -0,21 |
| **AF25NES** | -0,17 | -1,03 | 0,2 |
| **AF27NES** | 0,41 | -0,61 | 1,05 |

**Results**

**Measurement Invariance Analyses**

Before conducting the SEM, we tested measurement invariance to establish whether the measurement model with four latent variables was invariant across the two groups under investigation (i.e., participants who evaluated faces of males’ candidates or females’ candidates). To this end, we compared the configural (baseline) model with the metric model, in which factor loadings were constrained to be equal across groups. To determine differences between models, at least two out of these three criteria had to be matched: ΔχSB2 significant at *p* < .05 (Satorra & Bentler, 2001), ΔCFI ≥ -.010, and ΔRMSEA ≥ .015 (Chen, 2007). Model comparisons indicated that metric invariance could be clearly established (ΔχSB2 = 5.207, Δdf = 4, p = .267, ΔCFI = -.002, ΔRMSEA = .004). Based on this, we could reliably proceed with analyses aimed at unraveling associations among study variables across the two groups of interest.
